# The Effect of Exercise on Nutritional Status and Body Composition in Hemodialysis: A Systematic Review

**DOI:** 10.3390/nu12103071

**Published:** 2020-10-08

**Authors:** Dimitra Rafailia Bakaloudi, Antonios Siargkas, Kalliopi Anna Poulia, Evangelia Dounousi, Michail Chourdakis

**Affiliations:** 1 Laboratory of Hygiene, Social & Preventive Medicine and Medical Statistics, School of Medicine, Faculty of Health Sciences, Aristotle University of Thessaloniki, 54124 Thessaloniki, Greece; dimitrabakaloudi@gmail.com (D.R.B.); antonis.siargkas@gmail.com (A.S.); 2 Department of Nutrition and Dietetics, Laiko General Hospital, 11527 Athens, Greece; lpoulia@gmail.com; 3 Department of Nephrology, Faculty of Medicine, School of Health Sciences, University of Ioannina, 45110 Ioannina, Greece; evangeldou@gmail.com

**Keywords:** chronic kidney disease, hemodialysis, physical activity, nutritional status, body composition, sarcopenia

## Abstract

Chronic kidney disease (CKD) is associated with aggravating factors which can affect both body composition and nutritional status. The purpose of the present systematic review was to investigate the potential effects of any physical activity on body composition or nutritional status among patients with stage 5 CKD undergoing hemodialysis (HD). A literature search on PubMed, Scopus, Web of Science, Google Scholar, and Cochrane was conducted and 14 randomized clinical trials were included. Skeletal muscle index and mid-arm muscular circumference increased after resistance exercise, and the results on body mass index, % body fat, and lean body mass varied. Serum albumin and C-reactive protein, in most cases, showed a slight increase and decrease, respectively. An improvement was also observed in body strength and overall performance status. The results suggest that physical activity can be beneficial for both the body composition and nutritional status of patients undergoing HD and can help in the prevention of sarcopenia. However, further research is needed mainly in the field of nutritional status.

## 1. Introduction

Chronic kidney disease (CKD) is a major health problem with an estimated global prevalence of 11–13% [1]. Chronic kidney disease progression is divided into five stages and patients in the last stage are characterized by a progressive kidney failure and the need of renal replacement therapy (RRT), i.e., hemodialysis (HD), peritoneal dialysis (PD) or transplantation [2]. In CKD, physiological alterations of metabolism and physiology of the body are present such as deterioration of renal function, uremia as well as electrolyte and mineral derangements [3]. Patients with end-stage renal disease (ESRD) have increased risk of cardiovascular diseases (CVDs) and subsequently higher mortality risk compared to healthy adults [4]. Moreover, in stage 5 CKD there is a higher prevalence of malnutrition, chronic inflammation and oxidative stress, anemia, vitamin D deficiency, insulin resistance, functional capacity deterioration, lean body mass (LBM) wasting, and cachexia [5,6,7,8]. Nutritional status seems to worsen in long dialysis periods, and this is associated with the high rate of muscle mass and fat wasting [9] as well as a decrease in health-related quality of life (HrQoL) [10,11]. Decreased nutrient intake, due to the fact of anorexia or even dietetic restrictions, is also a common problem of stage 5 CKD [12]. The aforementioned alterations seem to have a negative impact on nutritional status and negatively affect body composition in patients undergoing HD [8,13,14].

Muscle functionality in patients with stage 5 CKD has been found to be compromised in previous studies [15,16,17]. This can be associated with lower performance status, physical activity intolerance, and muscle weakness [17], factors that can contribute to a higher percentages of patients with CKD leading a sedentary lifestyle [17,18]. Sarcopenia, the loss of skeletal muscle mass and its functionality, is highly prevalent in patients with CKD and is strongly associated with higher morbidity and mortality [19]. Patients with sarcopenia progressively lose muscle mass and strength, whereas the degree of sarcopenia is associated with the stage of CKD, especially in men [20]. In older adults, where sarcopenia is even more frequent due to the impact of aging, lower physical activity, and more prevalent ESRD, sarcopenia is even more profound and most of the time is refractory [21]. According to recently published studies, sarcopenic obesity, i.e., the co-existence of sarcopenia and obesity, not only diminishes any potential benefit from obesity (described as “obesity paradox”) but leads to substantially worse outcomes [22,23,24,25].

Enhancing physical activity has shown a beneficial impact on improving body composition in healthy subjects [26]. However, in patients with CKD, the symptoms of anemia, vascular dysfunction (arterial stiffness), muscle abnormalities, chronic metabolic acidosis, and inflammation can induce protein degradation which is associated with exercise intolerance and sedentary behavior among this population [27,28,29]. Moreover, there is a growing interest of the effect of physical activity and the overall health in patients with CKD, as it is considered to be one of the best ways to preserve muscle mass in this population [30]. According to the current guidelines for patients with CKD, including patients undergoing HD, physical activity is not contraindicated; on the contrary, it is considered to act beneficially [31]. However, HD patients have to counteract the “obligatory” sedentary time during HD sessions, resulting in even lower physical activity levels, lower physical performance, and a lower HrQoL [32].

In previous systematic reviews, the beneficial effects of exercise on the physical health of patients undergoing HD [33,34,35,36,37,38] as well as on the QoL were illuminated [39,40,41,42,43,44,45]. In two studies by Lu et al. [46] and Molsted et al. [47], the positive effects of exercise on muscle mass and muscle strength in patients undergoing HD were also stressed out. Nevertheless, according to our knowledge, there are no published studies in which the total body composition and nutritional status of patients undergoing HD in relation to exercise have been examined.

## 2. Materials and Methods 

The current study is a systematic review of randomized clinical trials (RCTs). The PubMed, Scopus, Web of Science, Google Scholar, and Cochrane database searches were performed (up to 21 July 2020) according to the following main search string: ((physical activity) OR (exercise)) AND (hemodialysis OR (renal failure) OR (kidney failure)). Our systematic review was conducted according to the PRISMA (Preferred Reporting Items for Systematic Reviews and Meta-Analyses) statement [48] (detailed information can be found in Table S1) and the Protocol was electronically submitted in the Prospero Library (CRD42020181769).

**Figure 1:**
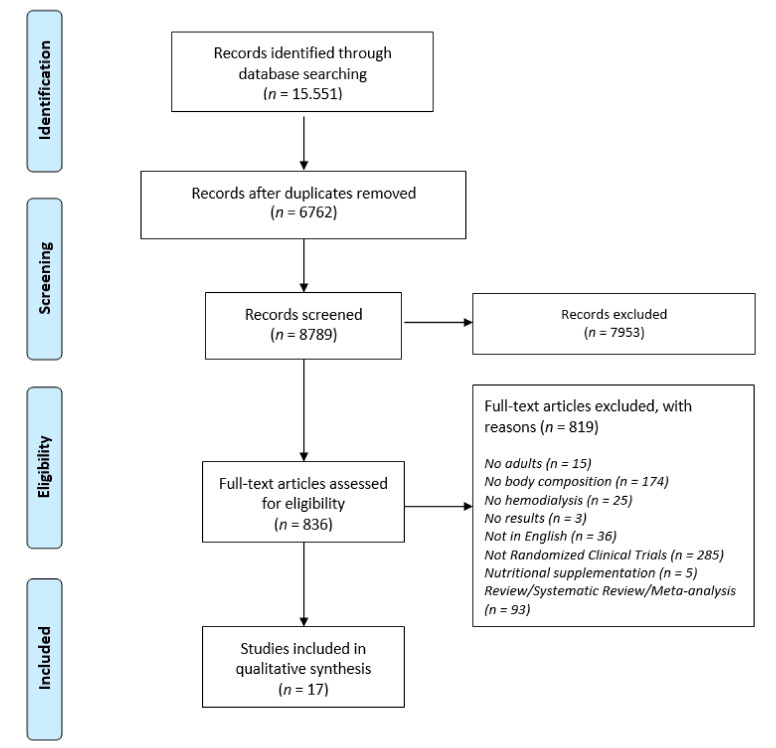
Preferred Reporting Items for Systematic Reviews and Meta-Analyses (PRISMA) flow diagram of the study selection process.

Initially, the output of our results (15.982 studies) was input into a reference database (EndNote X7 for Windows, Thomson Reuters) and duplicates were removed. Then, all titles and abstracts were examined for relevance by two researchers (DB and AS), and a third reviewer KAP was consulted when any doubts emerged. Overall, this resulted in the exclusion of 15.964 studies for not complying with the inclusion/exclusion criteria. The population of interest were patients undergoing HD with a duration of treatment of more than 3 months, >18 years old, and engaged in any type of physical activity. The control for the eligible RCTs were patients undergoing HD for more than 3 months, >18 years old but without performing significant physical activity at the baseline of intervention. Incomplete studies, studies with different control groups, studies not in the English language, or published before 2000 were excluded. As a result, 18 RCT studies [49,50,51,52,53,54,55,56,57,58,59,60,61,62,63,64,65,66] were characterized as acceptable; details regarding the eligibility process can be found in the flow diagram presented in Figure 1.

The main outcomes were the differences in nutritional status between the intervention and control groups using the examined serum albumin (sALB) and C-Reactive protein (CRP) and the effect of exercise on body composition using as the main evaluation variables: body mass index (BMI), mid-arm muscular circumference (MAMC) measurements, % of body fat (%BF), lean body mass (LBM), and skeletal muscle index (SMI) assessment. Muscle and fat mass (FM) evaluation methods included anthropometry (i.e., skinfold thickness and circumference measurements), computed topographies, dual-energy x-ray absorptiometry (DEXA), multiple-frequency bioelectrical impendence analysis (BIA) by various methods (i.e., body composition monitor, BCM; Fresenius Medical Care, Bad Homburg, Germany Maltron Inc., BioScan 920-2S Multifrequency Analyzer). The evaluation of total muscle strength, functionality, and the effects on performance status were reported as secondary outcomes. Assessment tools for hand grip strength (HGS) were a variety of dynamometers (Chatillon CSD 200 Dynamometer; Ametek Inc, Paoli, PA; CV, 9.4%, Lafayette Instrument, Lafayette, IN, T.K.K. 5401 GRIP D, Takei Science Instruments, Niigata, Japan, Cybex Inc., Ronkonkoma, NY, Takei TKK 5001 Tokyo, Japan, Jamar Hydraulic Hand Dynamometer) and the Wells Bench test. Exercise performance evaluations were conducted mainly by walking tests. The most common test was the 6 min walk test (6MWT) [67] or other similar tests. 

The quality evaluation of the eligible studies was conducted using the Cochrane Collaboration tool to assess risk of bias [68].

## 3. Results

The main characteristics of the included studies are presented in Table 1. In total, 945 patients undergoing HD were included in our study. The duration of physical activity intervention varied from 8 weeks to 2 years in eligible studies, and the frequency ranged from 2 to 4 times per week.

**Figure 2:**
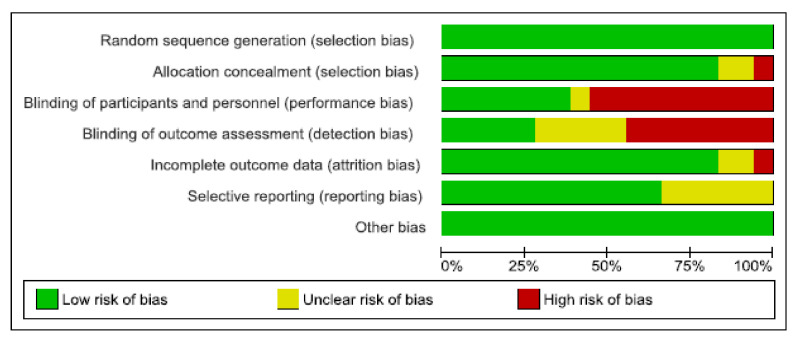
Risk of bias graph of included studies.

**Figure 3:**
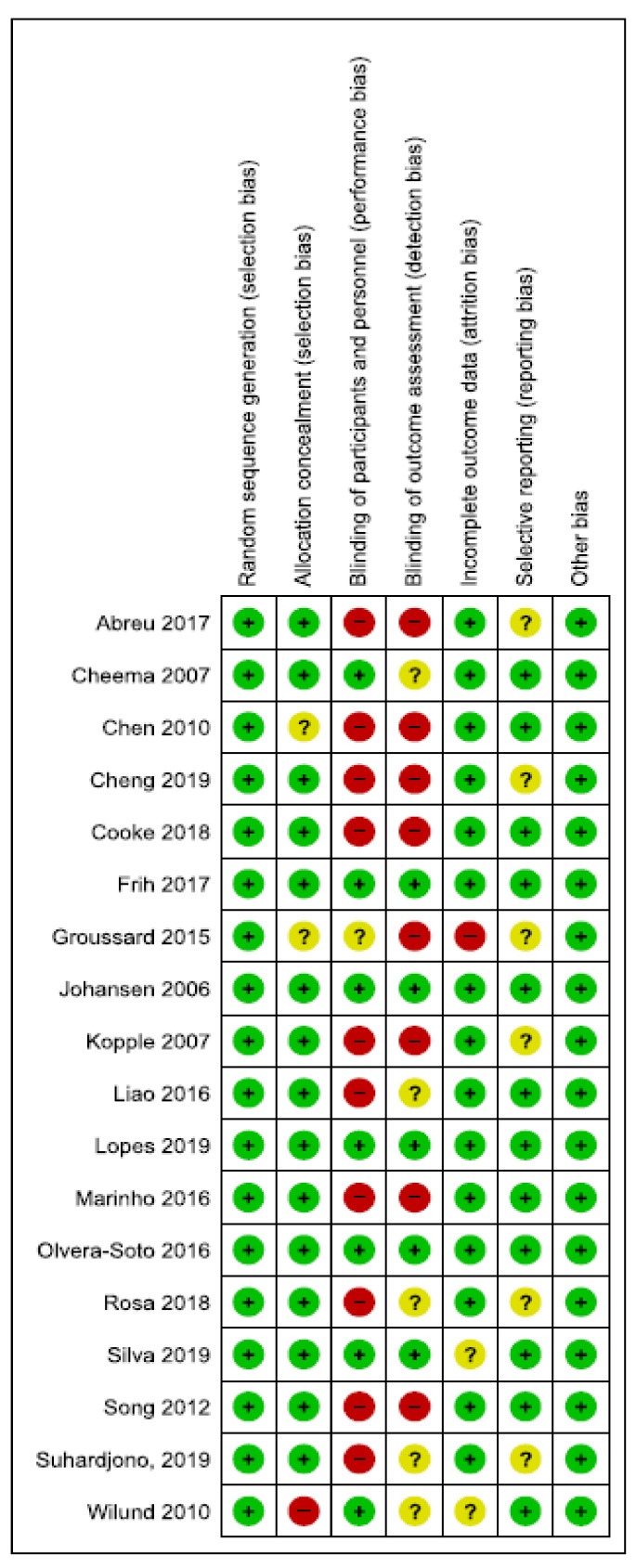
Risk of bias summary for included studies.

The assessment of risk of bias was conducted for all the 18 studies. In 11 studies, high or low unclear risk of bias was detected due to the absence of blinding of participants and/or outcome assessment [49,50,51,52,53,55,57,60,62,64,65]. Five studies were considered as low or unclear risk of bias because of selective reporting [49,55,57,62,65], and in only three studies high, or unclear risk of bias was detected due to the incomplete outcome of the data [55,63,66]. A summary of the assessment of risk of bias can be found in Figure 2 and Figure 3. A *p*-value <0.05 was considered of statistical significance. Statistical assessment of included studies included paired *t*-test, Wilcoxon or Mann–Whitney U test as appropriate and/or analysis of covariance (ANCOVA). These *p*-values show the level of significance of seen changes after the exercise parameters.

### 3.1. Body Mass Index (BMI)

Results regarding BMI changes among patients undergoing HD after the exercise intervention are presented in Table 2. These interventions (resistance training and/or pedaling) lasted for 12–18 weeks and BMI was calculated at baseline and at the end of the intervention. Body mass index was found to be increased in the intervention group in comparison to the non-active group (0.3 versus −0.1, 0.28 versus 0.2, 0.25 versus 0.03, 0.1 versus −0.3 accordingly) [50,53,55,62]. Similarly, Abreu et al. reported a significantly greater reduction of BMI in the control group versus the intervention group (−0.3 versus −0.1) [49]. In a study by Kopple et al. [57], a reduction in BMI was detected in all exercising groups irrespective of the type of training, (i.e., −0.3 m/kg^2^ in the endurance training group, −1.0 m/kg^2^ in the strength training group, and −0.2 m/kg^2^ in the combined group), whereas in the control group, BMI increased by 0.1 m/kg^2^ at the end of the intervention [57]. The same outcomes were reported by Marinho et al. [60], where resistance exercise led to a slight reduction (−0.1 m/kg^2^) of the BMI in the intervention group, while in the non-active group the reduction was found to be higher by 0.2 kg/m^2^ at the end of the intervention. Finally, in a study by Liao et al. [58], pedaling on a cycle ergometer during HD did not have any significant effects on BMI, while non-active patients reported a higher BMI (0.24 kg/m^2^) at the end of the study. On the contrary, pedaling in the Wilund et al. [66] study led to slight increase in BMI in the active patients in comparison to the non-active group in which a slight decrease was observed [66].

### 3.2. Mid-Arm, Waist, and Midthigh Circumferences

Increased MAMC was found in the physical active groups with patients following a program of resistance exercise for 30–50 min, 2–3 times/week in comparison to the non-active groups [49,50,61,64]. Moreover, in the studies by Abreu et al. [49] and Song et al. [64], waist circumference (WC) was found to be lower in the active subjects, whereas in non-active patients, WC increased [49,64]. In a study by Cheema et al. [50] an increase in midthigh circumference was reported among active patients, while the control group was found to have a slight decrease (+0.7 versus −0.3 cm, respectively) [50]. The results regarding circumferences are presented in Table 3.

### 3.3. Body Fat

The effect of exercise on the percentage of body fat (%BF) seemed to vary among the studies included in this systematic review, an effect that can be partially attributed to the type of exercise performed [51,55,56,57,59,60,61,62,64]. The results regarding %BF can be found in Table 4. In the studies by Johansen et al. [56] and Olvera-Soto et al. [61] there was a significantly higher increase in %BF in the exercise group versus the control group [56,61]. According to Rosa et al. [62] after 12 weeks of follow-up, %BF was reduced, but in non-active subjects the reduction was greater (mean difference −1.23% in the non-active versus −0.71% in the active group) [62]. Marinho et al. [60] reported a higher reduction in %BF in the intervention group compared to the controls (mean difference −0.9% versus −0.6%, respectively) [60]. The three types of exercise (endurance cycling, strength, and combined exercise) in the study by Kopple et al. [57] resulted in different outcomes for %BF [57]. Endurance cycling decreased %BF by ~0.5%, resistant exercise led to a slight increase in body fat by ~0.2%, and combined exercise decreased %BF by ~1.2% [57]. Similarly, in the Chen et al. [51] and Song et al. [64] studies, resistance exercise led to decrease in %BF in the active group in comparison to the control group in which an increase in %BF was observed [51,64]. Furthermore, in the study by Loppes et al. [59], the exercise intervention did not significantly affect %BF in the group following a high-intensity program, while in the group of moderate intensity, the reduction was slightly higher than the control group [59]. In the Groussard et al. [55] study, the low intensity of the resistance exercise did not have a statistical significant effect on %BF [55].

According to the study by Johansen et al. [56], lean body mass (LBM) decreased after 12 weeks of follow-up in both the intervention and control groups, but the decrease was greater in the intervention group [56]. In the study by Kopple et al. [57], the endurance cycling resulted in a slight reduction in LBM by ~−0.7 kg, while resistance training and combined exercise resulted in an increase in LBM by ~0.4 kg and ~0.5 kg, respectively [57]. Surprisingly, in the non-active group, LBM was higher by ~0.7 kg [57]. Similar outcomes recorded by Marinho et al. [60], where resistance exercise led to increased LBM compared to the initial measurement, but the increase was smaller in the intervention group compared to the controls (~1% versus 3%) [60]. On the contrary, in the studies by Chen et al. [51], Lopes et al. [59], and Rosa et al. [62], LBM increased after the intervention, while in the non-physically active subjects, LBM decreased or showed a slighter increase [51,59,62]. Similarly, in the Song et al. [64] study, an increase in the skeletal body mass was observed in the intervention group compared to the non-active group in which the skeletal body mass decreased [64]. Changes in LBM are presented in Table 5.

### 3.4. Skeletal Muscle Index

Two studies included in this systematic review examined changes in SMI, and both of them concluded that there was a significant improvement in SMI in the intervention group [59,65]. Resistance training with high-load led to a greater increase in SMI compared to the moderate-load group [59]. Moreover, in the aerobic exercise groups, the increase in SMI was greater than in the combined aerobic-resistance exercise group (0.15 versus 0.04, *p* < 0.05) [65]. The relevant results are presented in Table 6.

In the studies by Abreu et al. [49] and Wilund et al. [66], resistance and aerobic exercise did not significantly affect sALB, while CRP decreased compared to the non-active group [49,66]. Moreover, a slight increase in sALB and a reduction in CRP were observed by both Cheema et al. [50] and Kopple et al. [57], where resistance exercise [50] and cycling were the interventions, respectively [57]. Cycling in Liao et al. [58] increased sALB and decreased CRP after a 12 week intervention [58]. Resistance exercise resulted in a slight reduction in sALB, while combined exercise (i.e., cycling and resistance exercise) did not result in any significant effects on sALB [57]. In both groups (i.e., cycling and combined exercise), CRP increased [57]. Endurance-resistance training had an impact neither on sALB nor on CRP [54], while resistance-stretching exercise led to a greater decrease in CRP compared to the controls after a 2 year follow-up [52]. Increased CRP was also found in an intervention with resistance-exercise by Marinho et al. [60], and according to the data from Suhardjono et al. [65] there was a greater reduction in CRP only in subjects following combined aerobic-resistance exercise compared to the non-active patients undergoing HD [65]. The SALB and CRP changes are presented in Table 7.

### 3.5. Strength and Functionality Evaluation

In the majority of the studies included in our systematic review, strength increased after the intervention [50,51,54,56,59,61,62,64,65]. Resistance exercise significantly increased the total strength of active subjects, while in the control group there was a slight reduction of strength after the intervention period [50,51,56,64]. Endurance training also led to a notable increase in strength, measured by HGS measurement. More specifically, there was a mean increase of 12.4 N in the intervention group versus 0.7 N in the control group [54]. Non-significant differences in handgrip strength were recorded by Cooke et al. [53] and Suhardjono et al. [65] after pedaling [53,65] and in the moderate-intensity resistance exercise group by Lopes et al. [59]. These changes are presented in Table 8.

### 3.6. Performance Status

Regarding to the performance status, several studies illuminated a significant improvement in physically active subjects [50,52,53,54,55,62] as can be seen in Table 9. Assessment tools used for the evaluation of performance status were walking tests with modifications in distance and time. In the majority of the studies, the 6MWT method was used. Significant ameliorations were observed mainly after resistance exercise [50,52,54,62], while in the aerobic intervention group, an improvement was noted but at a lower grade [53,65]. However, in the study by Johansen et al. [56], resistance exercise led to a smaller improvement in gait speed compared to the non-active group, (2.7 cm/s versus 6 cm/s, respectively) [56].

## 4. Discussion

The aim of our systematic review was to investigate the effects of exercise on the nutritional status and body composition among patients undergoing HD. According to our systematic review, physical activity in patients undergoing HD resulted in beneficial outcomes, i.e., improved muscle strength and muscle mass, better performance status, increased Alb, and decreased CrP. Regarding the effects of physical activity on BMI, the findings were inconclusive. A decrease in BMI is not always desirable for patients undergoing HD, as in most cases it is difficult to identify if this reduction is associated with a decrease in LBM or in FM. According to studies including measurements of MAMC, there was a significant increase of this parameter in the physically active groups following resistance exercise. This improvement advocates an increase in muscle mass. Therefore, the reduction in BMI that was found was mostly accompanied by an increase in MAMC which means a preservation of muscle mass, with a relevant reduction in FM. These results are in concordance with studies including measurements of muscle mass by BIA and the calculation of SMI in which muscle mass as well as SMI increased after resistance exercise and were found to have a tendency to increase in the aerobic training group [46,69].

The results regarding body composition changes were inconclusive. Aerobic exercise and especially cycling seemed to reduce %BF [57]. On the other hand, results from our review could not significantly correlate physical activity with changes in LBM between intervention and control groups. The fact that the %BF results were unclear could explain similar uncertainty in LBM changes since the latter is defined as the difference between total body weight and body fat weight. Moreover, LBM estimation is influenced by fluids’ balance in the body, which in HD patients is heavily modified as a result of the fluid management between the HD sessions and the intradialytic fluid management.

One of the most commonly used laboratory markers for the evaluation of nutritional status is sALB [70,71]. However, sALB, as a nutritional parameter is characterized by a low specificity due to the fact of its long half-time, lasting approximately 20 days [72]. Therefore, nutritional status cannot be assessed solely by sALB [73]. Inflammation is also a significant factor influencing nutritional status and at the same time affecting the levels of sALB [73,74]. A variety of factors increase inflammation status in patients undergoing HD, i.e., alterations in gut microbiota, vascular disorders, and immunosuppression [74,75,76]. Therefore, the evaluation of both sALB and CRP can be used for the first approach of the overall nutritional and inflammatory status of patients undergoing HD [74]. Regarding the effect of physical activity on sALB and CRP, in the majority of studies, resistance exercise decreased CRP and slightly increased sALB, whereas aerobic exercise resulted in a reduction in sALB and an increase in CRP. A possible explanation of the irregularity of the results is the limited intervention time which could possibly mask the positive effects on sALB due to its long half time.. Nevertheless, in the study by Cheng et al. [52], with an intervention of resistance exercise lasting for 2 years, there was a slight increase in sALB and a decline in CRP. In the Cheema et al. [50] study, the protein catabolic rate showed a slight increase in the active group compared to passive group in which a slight decrease was observed (0.02 ± 0.31 g/kg/d versus −0.04 ± 0.17 g/kg/d). In the same study, a mini-nutritional assessment (MNA) was used in order to assess the risk of malnutrition and showed that the score was better in the passive group after the intervention [50]. On the contrary, in the Frih et al. [54] study in which the MNA was also performed, it was observed that the nutritional status was ameliorated after the intervention of resistance exercise [54]. Specifically, at baseline in the passive and intervention group, the risk of malnutrition was 85.7% and 70% accordingly, and in the final assessment the risk of malnutrition in the intervention group was 23.8% compared to an 85% risk for the passive group [54]. Further research is needed in this field in order to reach to safe conclusions due to the fact that nutritional and inflammation status can be affected both negatively and positively by lifestyle and nutritional parameters, i.e., smoking and overall food quality [10]. According to the existing studies, only in one study was pre-albumin, which can better reflect nutritional status, recorded [77]. A significant increase in pre-albumin was observed after resistance training compared to non-active patients [77]. Chan et al. [78] and Zhang et al. [79] found preliminary evidence that resistance training can reduce malnutrition and no significant improvement in nutrient intake, respectively.

According to our results, physical activity, especially resistance exercise, has a beneficial effect on body strength as measured by HGS dynamometry. Both upper and lower body mass strength were found to be increased at the end of the intervention in the active group versus the non-active one. On the other hand, cycling did not result in any statistically significant change in muscle strength. All types of exercise resulted in improvements in performance status, but resistance exercise seems to be the superior. However, we cannot ignore the fact that aerobic exercise is associated with improved cardiorespiratory function, lipid profile improvement, and an overall improvement in mental health and QoL [42]. Therefore, combined exercise could be the ideal choice for HD patients, providing a combination of positive results in many aspects of the patients’ lives. According to the recently revised criteria for the diagnosis of sarcopenia, muscle strength is the first alarming sign of sarcopenia, followed by the reduction of muscle mass [80]. Skeletal muscle mitochondrial dysfunction as well as reduction of muscle mitochondria are more prevalent in patients with CKD and could provide an explanation of the higher prevalence of sarcopenia in this population [81,82]. Mitochondria dysfunction plays an important role in inflammation and oxidative stress and, therefore, contributes to the pathogenesis of atherosclerosis and CVD [83,84]. According to Balakrishnan et al. [85], resistance exercise seems to act protectively by increasing the biogenesis and restoration muscle mitochondria in patients with CKD [85]. Therefore, the improvement in functional tests and the performance status after the analysis of the included RCTs in our study, especially of studies with interventions of resistance exercise protocols, could be a guide towards interventions that could prevent sarcopenia in this population [80].

Moreover, two recently published studies in which performance status [86] and strength [87] were examined concluded that the heterogeneity and the indistinct bias of the existing studies cannot lead to general conclusions [86,87].

The significant strengths of our systematic review are the careful selection of included studies, where all of them were randomized and had a similar control group. Moreover, most of the physical activity interventions were intradialytic, i.e., during the HD session and, therefore, physical activity was supervised during the entire time, had a specific duration and frequency (2–3 times per week), and was not omitted or skipped. Some limitations in the present study are that the assessment tools for body composition were different, and this could produce a bias of the results. The remarkable variability observed in the protocols and duration of training programs may partly explain the inconclusive evidence for some of the parameters. Moreover, serum prealbumin, which could be a more sensitive nutritional marker, was not assessed and, therefore, it could not be evaluated. Moreover, as nutritional intake was recorded in none of the RCTs, no conclusions can be derived about the possible effect of nutritional parameters in combination with exercise in patients undergoing HD.

## 5. Conclusions

Physical activity, in particular resistance exercise, seems to influence the body composition of patients undergoing HD. Increased MAMC and SMI were observed, whereas the results regarding %BF, LBM, and BMI were not clear. Resistance exercise may also control inflammation in patients undergoing HD, something that can have beneficial effects on lowering nutritional risks and/or malnutrition, but further research is needed in this field. Finally, performance status and strength (i.e., muscle functionality) can be improved in physically active patients undergoing HD, an effect with an undeniably positive impact on the QoL of all these patients. However, it needs to be noted that the existing evidence is insufficient to prove significant beneficial effects of exercise training on body composition and markers of nutritional/inflammation status mainly due to the high heterogeneity of protocols (in duration and type of exercise programs) of the existing clinical studies.

## Figures and Tables

**Table 1:** Characteristics of included Studies.

Identity	Exercise Type	Strength Assessment Tool	Performance Status Assessment	Body Composition Assessment Tool	Participants Exercise Group	Female/Male Exercise Group	Mean Age Exercise Group (Years)	Participants Control Group	Female/Male Control Group	Mean Age Control Group (Years)
Abreu et al. [49]	Resistance exercise 3 times/week for 12 weeks	N/A	N/A	Circumferences and skinfold thickness	25	54.5%/45.5%	45.7 ± 15.2	19	61.5%/38.5%	42.5 ± 13.5
Cheema et al. [50]	Resistance exercise 3 times/week for 12 weeks	HGS ^1^	6MWT	Computed tomography and standard protocols by dietician	24	7/17	60.0 ± 5.3	25	8/17	65.0 ± 12.9
Chen et al. [51]	Resistance exercise 2 times/week for 24 weeks	ΝA	NA	DEXA	22	10/12	71.1 ± 12.6	22	11/11	66.9 ± 13.4
Cheng et al. [52]	Resistance exercise 3 times/week for 2 years	N/A	6MWT	N/A	67	28/39	54.6 ± 12.6	65	25/40	55.8 ± 11.98
Cooke et al. [53]	Aerobic exercise 3 times/week for 16 weeks	HGS ^2^	6 m course as quickly as possible	N/A	10	3/7	58.2 ± 17.2	10	3/7	52.5 ± 15.4
Frih et al. [54]	Resistance training 4 times/week for 16 weeks	HGS ^3^	6MWT	N/A	21	0/21	64.2 ± 3.4	20	0/20	65.2 ± 3.1
Groussard et al. [55]	Aerobic exercise 3 days/weak for 3 months	NA	6MWT	DEXA	8	3/5	66.5 ± 4.6	10	3/7	68.4 ± 3.7
Johan-sen et al. [56]	Resistance exercise 3 times/week for 12 weeks	HGS ^4^	Walking 6 m at their usual pace and as fast as possible	DEXA	20	8/12	54.4 ± 13.6	20	6/14	56.8 ± 13.8
Kopple^,^ et al. [57] *	Aerobic exercise 3 times/week for 18 weeks	N/A	N/A	DEXA	10	4/6	45.9 ± 4.1	14	5/9	41.3 ± 3.3
Kopple et al. [57] *	Resistance training 3 times/week for 18 weeks	N/A	N/A	DEXA	15	6/9	46.0 ± 2.7	14	5/9	41.3 ± 3.3
Kopple et al. [57] *	Combined exercise 3 times/week for 18 weeks	N/A	N/A	DEXA	12	5/7	42.7 ± 3.8	14	5/9	41.3 ± 3.3
Liao et al. [58]	Aerobic exercise 3 times/week for 12 weeks	N/A	6MWT	DEXA	20	12/8	62 ± 8	20	11/9	62 ± 9
Lopes et al. [59] #	Resistance exercise (moderate load) 3 times/week for 12 weeks	HGS	N/A	DEXA	14	6/8	48.1 ± 10.8	20	1/13	56.9 ± 12.4
Lopes et al. [59] #	Resistance exercise (heavy load) 3 times/week for 12 weeks	HGS	N/A	DEXA	16	7/9	56.2 ± 12.5	20	1/13	56.9 ± 12.4
Marinho et al. [60]	Resistance exercise 3 times/week for 8 weeks	N/A	N/A	BCM	6	3/3	71.5 (58.5–87.2) *	7	4/3	76.0 (59.0-83.0) *
Olvera-Soto et al. [61]	Resistance exercise 2 times/week for 12 weeks	HGS ^5^	N/A	Circumferences and skinfolds	30	16/14	28.5 (23–46.5) *	31	12/2019	29 (19–38) *
Rosa et al. [62]	Resistance exercise 3 times/weeks for 12 weeks	Wells Bench test	6MWT	DEXA	28	8/2020	54.5 ± 11.97	24	9/15	57.10 ± 16.20
Silva et al. [63]	Aerobic exercise 3 times/week for 12 weeks	N/A	N/A	N/A	14	7/7	50 ± 17.2	14	6/8	58 ± 15.0
Song et al. [64]	Resistance exercise 3 times/week for 12 weeks	HGS ^6^	N/A	InBody s10^7^	20	12/8	52.1 ± 12.4	20	8/12	54.6 ± 10.1
Suhardjono^c^ et al. [65] @	Aerobic exercise 2 times/week for 12 weeks	HGS ^6^	walk 4 m back and forth for an 8 m distance	BIA	42	14/28	49.8 ± 11.7	39	21/18	50.5 ± 10.8
Suhardjono^c^ et al. [65] @	Combined exercise 2 times/week for 12 weeks	HGS ^6^	walk 4 m back and forth for a total distance of 8 m	BIA	39	21/18	50.5 ± 10.8	39	21/18	50.5 ±10.8
Wilund et al. [66]	Aerobic exercise 3 days/week for 4 months	N/A	N/A	N/A	7	4/3	60.8 ± 3.2	8	5/3	59.0 ± 4.9

N/A: not applicable; 6MWT: 6 min walk test; BC: body composition; DEXA: dual-energy X-ray absorptiometry, BIA: bioimpedance analysis (Maltron Inc., BioScan 920 2S Multifrequency Analyzer); BCM: body composition monitor; HGS: handgrip strength; ^*,#,@^ same control group; ^1^ isometric digital dynamometer (Chatillon CSD 200; Dynamometer; Ametek Inc, Paoli, PA; CV, 9.4%); ^2^ hand dynamometer (Lafayette Instrument, Lafayette, IN); ^3^ dynamometer (T.K.K. 5401 GRIP D, Takei Science; Instruments, Niigata, Japan); ^4^ computerized dynamometer (Cybex Inc., Ronkonkoma, NY); ^5^ Analogue Handgrip Dynamometer Takei TKK 5001 Tokyo, Japan; ^6^ Jamar Hydraulic Hand Dynamometer; ^7^ Biospace, Seoul, Korea. Values are presented as: mean ± SD. * Median (interquartile range).

**Table 2:** Results on BMI after intervention.

Identity	Exercise Group Before	Exercise Group After	Exercise Group Change	Control Group Before	Control Group After	Control Group Change	*p*-Value
Abreu et al. [49]	23.9 ± 4.7	23.8 ± 4.5	Not reported	24.4 ± 4.8	24.1 ± 4.9	Not reported	>0.05
Cheema et al. [50]	27.0 ± 6.0	Not reported	0.3 ± 0.5	28.0 ± 5.7	Not reported	0.1 ± 0.5	0.02
Cooke et al. [53]	25.6 ± 4.3	Not reported	0.28 (−0.23–0.95)	27.2 ± 6.1	Not reported	0.20 (−0.03–0.45)	0.485
Groussard et al. [55]	29.4 ± 2.1	29.5 ± 1.9	Not reported	26.5 ± 1.8	26.2 ± 1.9	Not reported	>0.05
Kopple et al. [57] a	26.9 ± 1.9	26.6 ± 1.8	Not reported	24.9 ± 1.1	25.1 ± 1.2	Not reported	>0.05
Kopple et al. [57] b	28.7 ± 2.5	27.7 ± 2.5	Not reported	24.9 ± 1.1	25.1 ± 1.2	Not reported	>0.05
Kopple et al. [57] c	26.2 ± 1.5	26.0 ± 1.5	Not reported	24.9 ± 1.1	25.1 ± 1.2	Not reported	>0.05
Liao et al. [58]	22.9 ± 3.3	22.96 ± 3.36	Not reported	23.67 ± 4.16	23.91 ± 5.27	Not reported	0.054
Marinho et al. [60]	28.5 (21.1–35.8)	28.4 (21.8–36.2)	Not reported	28.4 (20.8–35.2)	28.6 (23.6–35.2)	Not reported	>0.05
Rosa et al. [62]	26.4 ± 4.48	26.6 ± 4.44	Not reported	25.54 ± 3.95	25.5 ± 4.03	Not reported	0.752
Wilund et al. [66]	30.1 ± 2.4	30.3 ± 2.5	Not reported	29.0 ± 2.0	28.3 ± 1.8	Not reported	<0.05

^a^ Same control group; variables displayed as mean ± SD, median (interquartile range). a: Resistance exercise; b: combined exercise; c: aerobic exercise. a,b,c: same control group.

**Table 3:** Results on MAMC.

Identity	Exercise Group Before	Exercise Group After	Exercise Group Change	Control Group Before	Control Group After	Control Group Change	*p-*Value
Abreu et al. [49]	32.3 ± 14.6	33.9 ± 14.7	Not reported	35.6 ± 12.4	34.9 ± 15.2	Not reported	>0.05
Cheema et al. [50]	30.1 ± 4.0	Not reported	0.4 ± 1.4	30.1 ± 4.0	Not reported	−0.6 ± 0.9	0.004
Olvera-Soto et al. [61]	23.4 (20.3–25.4)	24.1 (20.3–26.5)	2.15 (−0.25 to 4.84) *	22.6 (19.7–25.2)	22.5 (19.6–25.5)	0.67 (−1.35 to 2.87) *	<0.01
Song et al. [64]	23.4 ± 1.4	23.5 ± 1.4	0.1 ± 0.7	23.7 ± 2.7	23.8 ± 2.6	0.0 ± 0.6	0.747

Variables displayed as the mean ± SD, median (interquartile range); * percentage (interquartile range).

**Table 4:** Effects on Body Fat.

Identity	Exercise Group Before	Exercise Group After	Exercise Group Change	Control Group Before	Control Group After	Control Group Change	*p-*Value
Chen et al. [51] (%)	31.3 ± 10.4	29.6 ± 9.8	Not reported	30.8 ± 11.2	33.1 ± 10.1	Not reported	0.9
Groussard et al. [55] (%)	32.2 ± 3.1	32.4 ± 3.2	Not reported	27.2 ± 2.7	27.3 ± 2.8	Not reported	>0.05
Johansen et al. [56] (kg)	22.4 ± 11.3	24.5 ± 11.1	2.2 ± 2.9	21.3 ± 11.9	21.4 ± 12.1	0.2 ± 1.6	0.05
Kopple et al. [57] a (%)	27.3 ± 3.0	26.8 ± 3.4	Not reported	24.3 ± 2.5	25.1 ± 2.6	Not reported	<0.01
Kopple et al. [57] b (%)	23.5 ± 2.6	23.7 ± 2.6	Not reported	24.3 ± 2.5	25.1 ± 2.6	Not reported	<0.01
Kopple et al. [57] c (%)	28.3 ± 2.6	27.1 ± 2.8	Not reported	24.3 ± 2.5	25.1 ± 2.6	Not reported	<0.01
Lopes et al. [59] A (kg)	20.0 ± 2.5	19.9 ± 2.5	Not reported	24.7 ± 2.1	24.6 ± 2.1	Not reported	0.69
Lopes et al. [59] B (kg)	23.7 ± 2.3	23.3 ± 2.3	Not reported	24.7 ± 2.1	24.6 ± 2.1	Not reported	0.69
Marinho et al. [60] (kg)	47.4 (33.6–48.8)	46.8 (35.0-48.6)	Not reported	53.0 (42.1–54.8)	52.1 (45.5–55.3)	Not reported	>0.05
Olvera-Soto et al. [61] (%)	16 (12.2–21.1)	16.8 (13.1–20.3)	5.43 (0.0 to 5.21) *	14 (9.4–18.3)	14.3 (11.3–18.8)	0.42 (−13.2 to 7.97) *	0.03
Rosa et al. [62] (%)	23.8 ± 9.21	23.10% ± 8.40	Not reported	23.15 ± 8.98	21.92 ±8.81	Not reported	0.619
Song et al. [64] (%)	27.5 ± 9.4	26.0 ± 8.6	−1.5 ± 3.7	26.0 ± 9.3	27.2 ± 8.9	1.2 ± 3.8	0.020

^a,b^ Same control group; variables displayed as the mean ± SD, median (interquartile range); * percentage (interquartile range). a: Resistance exercise; b: Combined exercise; c: Aerobic exercise. A: Resistance exercise (moderate load); B: Resistance exercise (heavy load). a, b, c: same control group. A, B: same control group 3.4. Lean body mass.

**Table 5:** Effects on Lean Body Mass (kg).

Identity	Exercise Group Before	Exercise Group After	Exercise Group Change	Control Group Before	Control Group After	Control Group Change	*p-*Value
Chen et al. [51]	45.8 ± 8.9	47.9 ± 9.9	Not reported	47.8 ± 9.0	46.3 ± 8.7	Not reported	0.5
Johansen et al. [56]	47.5 ± 12.3	47.1 ± 11.2	−0.3 ± 3.0	48.4 ± 8.2	48.2 ± 8.8	−0.1 ± 1.6	0.66
Kopple et al. [57] a	52.1 ± 0.28	51.4 ± 0.27	Not reported	47.7 ± 0.27	48.4 ± 0.26	Not reported	>0.05
Kopple et al. [57] b	47.3 ± 0.26	47.7 ± 0.27	Not reported	47.7 ± 0.27	48.4 ± 0.26	Not reported	>0.05
Kopple et al. [57] c	48.0 ± 0.33	48.5 ± 0.32	Not reported	47.7 ± 0.27	48.4 ± 0.26	Not reported	>0.05
Lopes et al. [59] A	39.1 ± 2.1	39.4 ± 2.2	Not reported	41.6 ± 1.8	41.5 ± 1.8	Not reported	0.60
Lopes et al. [59] B	41.6 ± 0.8	41.9 ± 0.8	Not reported	41.6 ± 1.8	41.5 ± 1.8	Not reported	0.60
Marinho et al. [60] (%)	34.7 (32.3–53.3)	35.7 (32.8–50.3)	Not reported	24.4 (18.9–39.0)	27.4 (23.8–34.0)	Not reported	>0.05
Rosa et al. [62]	46.55 ± 9.03	47.55 ± 9.49	Not reported	43.48 ± 8.02	44.04 ± 8.23	Not reported	0.277
Song et al. [64] (kg) *	21.4 ± 3.6	22.2 ± 3.7	0.8 ± 1.0	22.8 ± 5.3	22.5 ± 5.2	−0.3 ± 1.1	0.002

^a,b^ Same control group, * skeletal body mass, variables displayed as the mean ± SD, median (interquartile range). a: Resistance exercise; b: Combined exercise; c: Aerobic exercise. A: Resistance exercise (moderate load); B: Resistance exercise (heavy load). a, b, c: same control group. A, B: same control group.

**Table 6:** Effects on SMI (kg/m^2^).

Identity	Exercise Group Before	Exercise Group After	Exercise Group Change	Control Group Before	Control Group After	Control Group Change	*p*-Value
Lopes et al. [59] a	6.4 ± 1.2	6.6 ± 1.2	Not reported	6.8 ± 1.03	6.6 ± 1.1	Not reported	<0.01
Lopes et al. [59] b	6.7 ± 1.2	6.8 ± 1.1	Not reported	6.8 ± 1.03	6.6 ± 1.1	Not reported	<0.01
Suhardjono et al. [65] A	Males 10.4 ± 1.16 Females 9.77 ± 0.58	Not reported	0.15 (−2.11–2.89)	Males 9.92 ± 1.46 Females 9.79 ± 1.17	Not reported	0.01 (−6.14–7.33)	>0.05
Suhardjono et al. [65] B	Males 9.92 ± 1.46 Females 9.79 ± 1.17	Not reported	0.04 (−0.85–4.19)	Males 9.92 ± 1.46 Females 9.79 ± 1.17	Not reported	0.01 (−6.14–7.33)	>0.05

^a,b^ Same control group; variables displayed as the mean ± SD or median (min–max). a: Resistance exercise (moderate load); b: Resistance exercise (heavy load). a, b: same control group. A: Combined exercise; B; Aerobic exercise. A, B: same control group. 3.6. Serum Albumin and C-Reaction Protein

**Table 7:** Changes in sALB (g/dl) and CRP (mg/L).

Identity	Parameter	Exercise Group Before	Exercise Group After		Exercise Group Change	Control Group Before	Control Group After	Control Group Change	*p-*Value
Abreu et al. [49]	sALB	4.3 ± 0.3	4.3 ± 0.3		Not reported	4.2 ± 0.2	4.2 ± 0.2	Not reported	
	CRP	7.7 ± 6.0	5.8 ± 4.4			8.54 ± 4.2	8.4 ± 7.5		>0.05
Cheema et al. [50]	sALB	3.45 ± 0.31	Not reported		0.03 ± 0.24	3.36 ± 0.79	Not reported	0.01 ± 0.24	0.45
	CRP	0.78 ± 0.60			−0.08 ± 0.37	0.72 ± 0.55		0.24 ± 0.37	0.02
Cheng et al. [52]	sALB	4.05 ± 0.27	4.09 ± 0.18		Not reported	3.96 ± 0.32	4.02 ± 0.36	Not reported	0.747
	hs-CRP	0.25 (0.08–0.37)	0.15 (0.06–0.55)			0.28 (0.16–0.43)	0.26 (0.15–0.52)		
Frih et al. [54]	sALB	3.96 ± 0.35	0.40 ± 0.26		Not reported	3.99 ± 0.37	4.04 ± 0.37	Not reported	
	CRP	4.1 ± 1.3	4.1 ± 1.3			4.1 ± 1.1	4.0 ± 1.4		>0.05
Kopple et al. [57] a	sALB	3.7 ± 0.1	3.8 ± 0.1		Not reported	3.9 ± 0.1	3.9 ± 0.1	Not reported	>0.05
	CRP	4.5 ± 1.5	2.5 ± 0.6			2.1 ± 0.4	2.8 ± 0.8		
Kopple et al. [57] b	sALB	3.9 ± 0.1	3.8 ± 0.1		Not reported	3.9 ± 0.1	3.9 ± 0.1	Not reported	
	CRP	3.5 ± 0.8	4.2 ± 1.3			2.1 ± 0.4	2.8 ± 0.8		>0.05
Kopple et al. [57] c	sALB	3.8 ± 0.1	3.8 ± 0.1		Not reported	3.9 ± 0.1	3.9 ± 0.1	Not reported	
	CRP	4.6 ± 1.4	5.8 ± 2.1			2.1 ± 0.4	2.8 ± 0.8		>0.05
Liao et al. [58]	sALB	3.89 ± 0.33	4.16 ± 0.30		Not reported	4.00 ± 0.35	4.01 ± 0.42	Not reported	
	Hs-CRP	1.25 ± 2.01	0.78 ± 0.83			1.24 ± 2.04	1.23 ± 0.21		<0.05
	CRP	0.7 ± 0.33	0.6 ± 0.20			1.2 ± 0.97	1.5 ± 0.89		<0.01
Wilund et al. [66]	CRP	5.2 ± 0.78		4.9 ± 0.69	Not reported	6.2 ± 0.22.	6.0 ± 0.67	Not reported	<0.05
	sALB	3.9 ± 0.14		3.9 ± 0.15		3.8 ± 0.09	3.8 ± 0.06		

sALB: serum Albumin, CRP: C-Reactive Protein, Hs-CRP: high sensitivity C-Reactive Protein; ^a^ same control group; variables displayed as the mean ± SD or the median (interquartile range). a: Resistance exercise; b: Combined exercise; c: Aerobic exercise. a, b, c: same control group

**Table 8:** Results of the strength evaluation.

Identity	Parameter	Exercise Group Before	Exercise Group After	Exercise Group Change	Control Group Before	Control Group After	Control Group Change	*p-*Value
Cheema et al. [50]	Total strength	98.1 ± 36.6	Not reported	15.2 ± 15.4	86.0 ± 33.8	Not reported	−2.4 ± 13.8	0.002
Chen et al. [51]	Knee extensors strength (kg)	11.4 ± 5.0	15.8 ± 5.0	Not reported	14.8 ± 6.0	12.1 ± 6.1	Not reported	0.08
Cooke et al. [53]	HGS	23.2 ± 10.5	Not reported	1.3 (−0.5, 6.5)	25.9 ± 13.8	Not reported	2.5 (−0.5, 4.0)	0.464
Frih et al. [54]	Handgrip force (N)	29.8 ± 6.0	37.4 ± 4.8	Not reported	29.3 ± 5.6	30 ± 5.2	Not reported	<0.05
Johansen et al. [56]	Knee extension 3RM (lb)	14.0 ± 8.4	22.6 ± 11.6	8.6 ± 6.9	19.2 ± 8.7	20.0 ± 9.1	0.8 ± 2.0	<0.0001
	Hip abduction 3RM (lb)	8.5 ± 5.2	15.4 ± 6.9	6.9 ± 5.0	11.8 ± 4.3	11.8 ± 5.9	−0.1 ± 2.5	<0.0001
	Hip flexion 3RM (lb)	7.6 ± 5.3	13.7 ± 6.8	6.1 ± 4.3	10.9 ± 4.5	11.4 ± 6.3	0.5 ± 2.7	<0.0001
Lopes ^a^ et al. [59] a	HGS (kg)	29.2 ± 10.2	32.1 ± 11.4	Not reported	25.3 ± 9.1	25.4 ± 9.9	Not reported	0.60
Lopes ^a^ et al. [59] b	HGS (kg)	30.0 ± 8.7	29.9 ± 10.1	Not reported	25.3 ± 9.1	25.4 ± 9.9	Not reported	0.60
Olveira-Soto et al. [61]	HGS (kg)	19.6 (11–28)	21.2 (13–32)	Not reported	19.8 (14-26)	17.8 (15-26)	Not reported	<0.01
Rosa et al. [62]	HGS (kg/strength)	65.7 ± 23.3	66.61 ± 22.22	Not reported	59.21 ± 20.66	58.52 ± 18.19	Not reported	0.213
Song et al. [64]	HGS (kg)	26.3 ± 8.5	28.7 ± 9.0	2.4 ± 2.8	26.2 ± 10.2	27.8 ± 11.8	1.6 ± 4.0	0.465
	Leg muscle strength (kg)	33.0 ± 15.3	37.3 ± 19.0	4.3 ± 8.7	34.8 ± 20.3	33.4 ± 19.5	−1.4 ± 7.0	0.027
Suhardjono ^b^ et al. [65] A	HGS (kg)	Males: 24.8 ± 9.19 Females: 14.6 ± 4.66	Not reported	−0.08 (−2.83–18.50)	Males: 22.1 ± 9.26 Females: 17.3 ± 8.27	Not reported	−0.1 (2.78)	>0.05
Suhardjono ^b^ et al. [65] B	HGS (kg)	Males: 21.6 ± 8.84 Females: 18.3 ± 5.45	Not reported	0 (−5.33–9.50)	Males: 22.1 ± 9.26 Females: 17.3 ± 8.27	Not reported	−0.1 (2.78)	>0.05

HGS: Handgrip strength; RM: repetition maximum; ^a,b^ same control group; variables displayed as the mean ± SD, median (interquartile range), median (min–max). a: Resistance exercise (moderate load); b: Resistance exercise (heavy load). a, b: same control group. A: Combined exercise; B; Aerobic exercise. A, B: same control group.

**Table 9:** Changes on performance status.

Identity	Test Used	Exercise Group Before	Exercise Group After	Exercise Group Change	Control Group Before	Control Group After	Control Group Change	*p*-Value
Cheema et al. [50]	6MWT (m)	496.0 ± 138.9	Not reported	19.6 ± 4.0	412.6 ± 138.9	Not reported	1.5 ± 23.7	0.16
Cheng et al. [52]	6MWT (m)	439.1 ± 85.5	490.5 ± 70.3	Not reported	460.3 ± 79.1	456.7 ± 94.1	Not reported	0.207
Cooke et al. [53]	6 m course as quickly as possible (m/s)	0.8 ± 0.2	Not reported	0.02 (−0.02, 0.11)	0.9 ± 0.3	Not reported	−0.11 (−0.17, 0.08)	0.158
Frih et al. [54]	6MWT (m)	420 ± 35.1	480.5 ± 31.9	Not reported	422.2 ± 26.6	415.6 ± 36.3	Not reported	<0.05
Groussard et al. [55]	6MWT (m)	406 ± 18	500 ± 30	Not reported	376 ± 20	406 ± 18	Not reported	<0.001
Johansen et al. [56]	Walking 6m at their usual pace and as fast as possible (gait speed-cm/s)	100.9 ± 35.5	103.5 ± 34.2	2.7 ± 17.3	99.8 ± 31.5	105.7 ± 31.1	6.0 ± 17.2	0.71
Rosa et al. [62]	6MWT (m)	506.1 ± 130.3	526.5 ± 126.2	Not reported	452.65 ± 169.19	469.4 ± 162.9	Not reported	0.277
Suhardjono et al. [65] a	Walk 4 m back and forth for a total distance of 8 m (m/s)	Males: 0.86 ± 0.25 Females: 0.81 ± 0.2	Not reported	0.08 ± 0.16	Males: 0.8 ± 0.25 Females: 0.81 ± 0.23	Not reported	0.07 ± 0.19	>0.05
Suhardjono et al. [65] b	Walk 4 m back and forth for a total distance of 8 m (m/s)	Males: 0.87 ± 0.19 Females: 0.85 ± 0.17	Not reported	0.10 ± 0.12	Males: 0.8 ± 0.25 Females: 0.81 ± 0.23	Not reported	0.07 ± 0.19	>0.05

6MWT: 6 min walk test; variables displayed as the mean ± SD or median (interquartile range). a: Combined exercise; b: Aerobic exercise. a, b: same control group.

## Supplementary Materials

The following are available online at https://www.mdpi.com/2072-6643/12/10/3071/s1, Table S1: Preferred Reporting Items for Systematic Reviews and Meta-Analyses (PRISMA) Checklist.

## Abbreviations

BCM	Body composition monitor
BF	Body fat
BMI	Body mass index
CKD	Chronic kidney disease
CRP	C-reactive protein
CVD	Cardiovascular diseases
DEXA	Dual-energy x-ray absorptiometry
ESRD	End-stage renal disease
FM	Fat mass
HD	Hemodialysis
HrQoL	Health related quality of life
HGS	Hand grip strength
LBM	Lean body mass
MAMC	Mid arm muscular circumference
MNA	Mini-Nutritional Assessment
PD	Peritoneal dialysis
RCT	Randomized clinical trial
RRT	Renal replacement therapy
sALB	Serum Albumin
SD	Standard deviation
SMI	Skeletal muscle mass
QoL	Quality of life
WC	Waist circumference
6MWT	6 min walk test
